# A randomized trial of the efficacy of artesunate and three quinine regimens in the treatment of severe malaria in children at the Ebolowa Regional Hospital, Cameroon

**DOI:** 10.1186/s12936-015-0948-0

**Published:** 2015-10-31

**Authors:** Daniel Ethe Maka, Andreas Chiabi, Valentine Ndikum, Dorothy Achu, Evelyn Mah, Séraphin Nguefack, Pamela Nana, Zakariaou Njoumemi, Wilfred Mbacham, Elie Mbonda

**Affiliations:** Department of Paediatrics, Faculty of Medicine and Biomedical Sciences, University of Yaounde I, Yaounde, Cameroon; Paediatric Unit, Yaounde Gynaeco-Obstetric and Paediatric Hospital, Yaounde, Cameroon; Department of Pharmacology, Faculty of Medicine and Biomedical Sciences, University of Yaounde I, Yaounde, Cameroon; Case Management Section, Cameroon National Malaria Control Programme, Yaounde, Cameroon; Paediatric Unit, Ebolowa Regional Hospital, Ebolowa, Cameroon; Department of Public Health, Faculty of Medicine and Biomedical Sciences, University of Yaounde I, Yaounde, Cameroon; Department of Biochemistry, Faculty of Medicine and Biomedical Sciences, University of Yaounde I, Yaounde, Cameroon

**Keywords:** Artesunate, Quinine, Efficacy, Severe malaria, Children, Cameroon

## Abstract

**Background:**

Severe malaria is a medical emergency with high mortality in children below 5 years of age especially in sub-Saharan Africa. Recently, quinine has been replaced by artesunate as the first-line drug in the treatment of severe malaria in Cameroon. No local data are yet available on the efficacy of artesunate with respect to the different quinine regimens used in this setting. This study was undertaken at the Ebolowa Regional Hospital (ERH), which is located in a region of perennial transmission of malaria.

**Methods:**

This was a randomized, open-label trial in children aged 3 months to 15 years, admitted in the hospital with severe malaria due to *Plasmodium falciparum* confirmed on microscopy after informed parental consent. Patients were randomized into four groups. Group 1 (ARTES) received parenteral artesunate at 2.4 mg/kg at H_0_, H_12_, H_24_ and then once daily; Group 2 (QLD) received a loading dose of quinine base at 16.6 mg/kg followed 8 hours later by an eight-hourly maintenance dose of 8.3 mg/kg quinine base; Group 3 (QNLD3) received 8.3 mg/kg quinine base every 8 hours; and, Group 4 (QNLD2) received 12.5 mg/kg quinine base every 12 h. All patients invariably received a minimum of 24 h parenteral treatment, then, oral drugs were prescribed. The endpoints were fever clearance time, time to sit unsupported, time to eat, parasite clearance time, and parasitaemia reduction rate at H24. Survival analysis was used to compare the outcomes.

**Results:**

One-hundred and sixteen patients completed the study: 29 in ARTES arm, 28 in QLD arm, 30 in QNLD3 arm, and 29 in QNLD2 arm. There was no major differences in baseline characteristics in the treatment groups. On analysis of endpoints, fever clearance time and parasite clearance time were significantly shorter for artesunate-treated patients than for quinine-treated patients. Parasitaemia reduction rate at H24 was also significantly higher for artesunate. Time to sit unsupported and time to eat were shorter with artesunate, but the difference was not statistically significant.

**Conclusion:**

Artesunate is more effective than quinine in the treatment of severe malaria in Cameroonian children.

## Background

Malaria control is a cornerstone in the reduction of child mortality and constitutes the fourth Millennium Development Goal [1]. To date, malaria remains a serious public health problem in many parts of the world, especially in sub-Saharan Africa. According to the World Health Organization (WHO), in 2012 there were an estimated 207 million reported cases of malaria and 627,000 malaria deaths globally. Approximately 80 % of the cases and 90 % of deaths occurred in the WHO African Region and 77 % were in children under 5 years of age [2]. Malaria remains the most widely spread disease in the world; populations living in sub-Saharan Africa have the highest risk of acquiring malaria [3].

In Cameroon, malaria is a major endemic parasitic disease and the first cause of morbidity and mortality. Children under 5 years old and pregnant women are the most vulnerable population groups. Malaria alone is responsible for 24 % of total deaths, 40–45 % of medical consultations and 30 % of hospital admissions in children under 59 months old [4].

The mortality of untreated severe malaria (particularly cerebral malaria) approaches 100 %. With prompt, effective anti-malarial treatment and supportive care, mortality falls to 15–20 % overall, making it a medical emergency [3]. In line with this, since 2010, WHO strongly recommends parenteral artesunate as the drug of choice and quinine as the second-line drug in the treatment of severe malaria in adults and children worldwide [3]. WHO currently recommends that a loading dose of 16.6 mg/kg body weight (bw) of quinine base (QB) be administered, followed 8 hours later by a maintenance dose of 8.3 mg/kg bw every 8 hours.

In 2013, the Cameroon National Malaria Control Programme (NMCP) revised its treatment directives and adopted the above WHO treatment regimens. The programme also recommended a non-loading dose regimen of 8.3 mg/kg bw of quinine base (QB) eight-hourly [5]. In some health institutions in the country, a two-daily administration of 12.5 mg/kg bw of QB is used due to feasibility and limited personnel. However, this latter regimen is not recommended by the NMCP.

Despite the fact that artesunate has been recommended for severe malaria, uptake of parenteral artesunate for the treatment of severe malaria is still slow in Cameroon due to conservative attitudes and possibly due to the lack of country-specific data. Therefore, this study was undertaken to assess the efficacy of artesunate with respect to other quinine regimens currently used in this context for the treatment of severe malaria in children in a hospital located in a malaria-endemic geographical region in south Cameroon.

## Methods

### Study area

This study was conducted in the paediatric unit of the Ebolowa Regional Hospital (ERH). This hospital is located in Ebolowa, which is the headquarters of the South Region of Cameroon and is the referral hospital in that region. It has a total capacity of 158 beds for an estimated population of 120,000 inhabitants. The paediatric unit itself has a capacity of 28 beds and is headed by a paediatrician assisted by a physician and ten nurses working in teams of two to three. The Ebolowa health district in which the hospital is located is a heavily malaria-infested area characterized by a stable, perennial malaria transmission.

### Study design

This was an open-label, randomized, clinical trial carried out from 1 September, 2013–31 March, 2014. Included in the study were all children with malaria aged 3 months to 15 years irrespective of sex, presenting with one or more signs of severity of malaria according to the 2013 Cameroon NMCP–adopted criteria (impaired consciousness, abnormal behaviour, convulsions, prostration, persistent vomiting, jaundice, hyperthermia, acute respiratory distress, clinical acidosis, haemoglobinuria, cardiovascular shock, dehydration, abnormal bleeding, severe anaemia, renal impairment, hypoglycaemia, and hyperparasitaemia) and having an initial positive parasitaemia to *Plasmodium falciparum.* Other aetiologies of the presenting symptoms were excluded and parents gave written informed consent. Excluded from the study were children who have had prior side-effects to either artesunate or quinine administration, severely malnourished children, and those who had a concomitant infection.

### Randomization and masking

Eligible patients were randomly assigned to receive either parenteral artesunate or one of the three quinine regimens. Using Kendall and Babington Smith random number table, an assistant not involved in the study performed the randomization in advance in blocks of 20 composed of five of each regimen. Treatment allocations were placed in numbered, opaque, sealed envelopes to which the investigator was blinded until a patient was admitted. On admission, each patient was attributed an envelope corresponding to his unique identification number.

### Procedure

Before recruitment of patients for the study, a pre-study training pilot study was carried out from 13 December, 2012–31 January, 2013. During the study period, any patient admitted for malaria in the paediatric unit with one or more signs of severity of malaria was immediately re-evaluated and anthropometric and vital parameters taken. An initial thin and thick blood film (at H_0_) was done to determine the *Plasmodium* species and the parasite load, respectively. The thin and thick films were stained in 10 % Giemsa solution for 10–15 min and read at ×100 after oil immersion. The parasite load was determined by counting the number of parasites in at least 20 oil immersion fields. Each slide was double-checked blindly and was considered to be negative if no parasites were detected in at least 20 oil immersion fields. The following investigations were also done systematically on admission: capillary glycaemia, a urine dipstick for children aged between 3 months and 5 years and a full blood count. A lumbar puncture was systematically done to patients with neurological symptoms to rule out meningitis. Chest X-ray, blood group and rhesus typing, blood urea nitrogen, creatinine, and bilirubin levels were done depending on the clinical presentation. After obtaining samples for investigation, treatment was initiated while awaiting the results of the blood film. If the initial film was negative, the child was excluded from the study and the initiated treatment was continued.

Patients were randomized into four groups. Those of the first group (ARTES) received 2.4 mg/kg body weight (bw) of parenteral artesunate at H_0_, H_12_, H_24_ and thereafter once daily. Each vial contained 60 mg anhydrous artesunic acid, which was dissolved in 1 ml of 5 % sodium bicarbonate (provided with the drug) and then mixed with 5 ml saline solution (provided with the drug) before injecting into an indwelling intravenous catheter. For the second group (QLD) the patients received a loading dose of 16.6 mg/kg bw of QB by intravenous (IV) infusion over 4 hours followed 8 hours after the start of the loading dose, with a maintenance dose of QB at 8.3 mg/kg bw over 4 hours every 8 hours. Patients in the third group (QNLD3) received 8.3 mg/kg bw of QB over 4 hours by IV infusion every 8 hours. Those in the fourth group (QNLD2) received 12.5 mg/kg bw of QB over 4 hours by IV infusion every 12 h. For the three quinine regimens, quinine was diluted in 10 ml/kg bw of 10 % dextrose. Clinical side-effects of the drugs were monitored during the treatment. Patients received a minimum of 24 h of parenteral treatment and exited from the protocol if they had clinically recovered and parasitaemia was negative. Thereafter, oral anti-malarials (artemether-amodiaquine or artemether-lumefantrine) were prescribed for 3 days to complete the treatment. Resuscitation and supportive management was done in accordance with WHO guidelines.

From the onset of treatment, the temperature was monitored every 6 hours until normalization; the state of consciousness was evaluated every 2 hours for the first 24 h then every 6 hours until normalization, using the Glasgow coma scale (GCS) or Blantyre coma scale (BCS) in children under 5 years of age. Parasitaemia was measured every 6 hours until it became negative and the time the child was able to sit unsupported and to eat if this was not possible on admission was also recorded.

### Endpoints

Endpoints of the study were clinical and parasitological outcome. The clinical outcome variables were: fever clearance time (FCT = time from the onset of treatment until rectal temperature went down to 37.5 °C for at least 24 h), coma recovery time ((CRT) time from the onset of treatment until the patient was fully conscious), time to sit unsupported, time to eat and time to exit from the protocol (=time from the onset of treatment until the patient had clinically recovered and was parasitaemia negative). The parasitological outcome variables were: parasite clearance time (PCT = time from the onset of treatment to the time of the first of two successive negative blood smears) and the parasite reduction rate 24 h after onset of treatment (PRR_24_).

### Statistical analysis

Data were coded as variables, introduced in Epi Info™ version 3.5.3 software and double-checked before analysis. Categorical variables were summarized in cross-tables; Chi-square test was used to compare the different proportions and Fisher’s exact test was used to test for associations two-by-two for cells less than five. Continuous variables were summarized into means and standard deviations and were compared by Kruskal–Wallis one-way analysis of variance (ANOVA). A two-group comparison (ARTES/QLD, ARTES/QNLD3 and ARTES/QNLD2) of the outcome variables was done and Student *t* test was used to compare the groups. Kaplan–Meier plots were determined for time to event outcomes and compared by Log-Rank test. A *p* value less than 0.05 was considered statistically significant.

### Ethics

Authorization from the Director of ERH was obtained before starting the study in the paediatric unit of the hospital. Ethical clearance was obtained from the Ethics Committee of the Faculty of Medicine and Biomedical Sciences. The study procedure and justification was explained to the parents and an informed written consent from the parents was obtained before enrolment of the children in the study. For confidentiality, code names were given to all patients. During the study, the work sheets were kept secret by the investigators from people not involved in the study, in order to respect confidentiality of collected data. The information provided was used for this study only and was not shared for any other purposes or projects. Blood films, capillary glycaemia, urine dipstick and artesunate ampoules were done at the expense of the investigators.

## Results

**Fig. 1:**
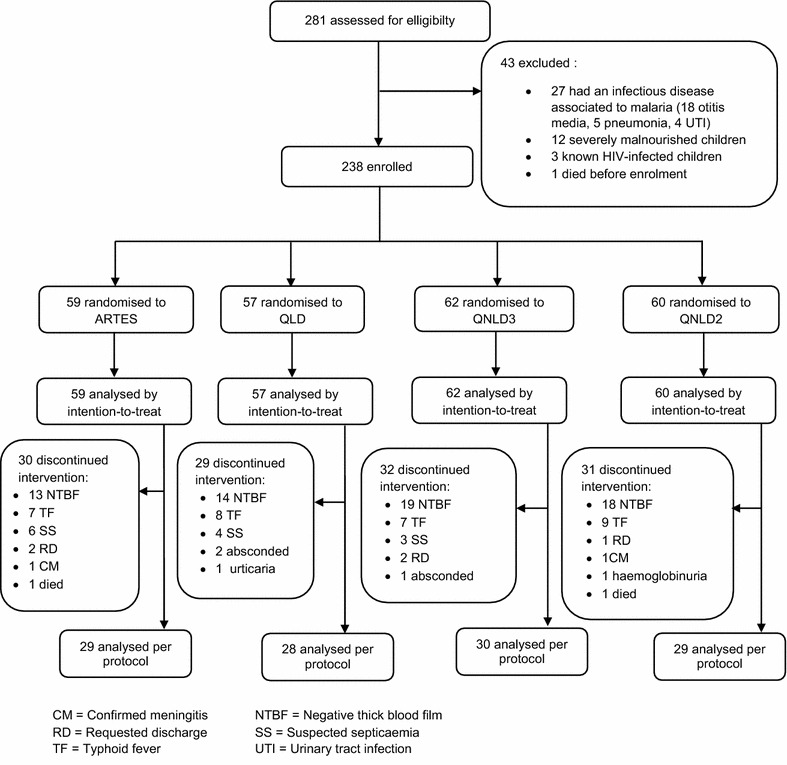
Trial profile

A total of 281 children admitted for severe malaria at ERH were assessed for eligibility. Following diligent scrutiny of the inclusion and exclusion criteria, 238 patients were enrolled in the study. Fifty-nine were randomized to the ARTES (artesunate) group, 57 to QLD (quinine loading dose) group, 62 to QNLD3 (quinine non-loading dose, three daily administration) group, and 60 to QNLD2 (quinine non-loading dose, two daily administration) group. Figure 1 shows the trial profile with participant flow.

Data were analysed primarily by intention-to-treat for the 238 patients enrolled in the study and secondarily per protocol for the remaining sub-set of patients who completed the treatment assigned (n = 116).

Clinical and laboratory parameters on admission did not significantly differ between the artesunate and the quinine groups (p > 0.05; Tables 1, 2).

**Table 1:** Clinical and paraclinical baseline characteristics on admission

	Regimens					p value
	ARTES (n = 59)	QLD (n = 57)	QNLD3 (n = 62)	QNLD2 (n = 60)	Total (n = 238)	
Age (months), mean ± SD	27.8 ± 23.0	34.0 ± 28.6	34.0 ± 28.5	38.0 ± 25.2	33.1 ± 24.6	0.08
Sex						
Female	33 (55.9 %)	32 (56.1 %)	34 (54.8 %)	26 (43.3 %)	125 (52.5 %)	0.43
Male	26 (44.1 %)	25 (43.9 %)	28 (45.2 %)	34 (56.7 %)	113 (47.5 %)	
Weight (kg), mean ± SD	12.6 ± 5.6	13.2 ± 4.7	12.7 ± 4.5	14.8 ± 7.4	13.3 ± 5.7	0.18
Temperature (°C), mean ± SD	39.3 ± 0.92	39.2 ± 0.92	39.3 ± 0.93	39.7 ± 0.86	39.3 ± 0.91	0.09
Heart rate (bpm), mean ± SD	143.4 ± 18.1	145.6 ± 17.5	140.1 ± 17.6	141.9 ± 18.0	142.7 ± 17.2	0.29
Respiratory rate (cpm), mean ± SD	44.3 ± 11.2	40.6 ± 9.7	40.3 ± 9.7	39.9 ± 8.6	41.3 ± 9.9	0.19
Coma depth (BCS), median	2 (n = 3)	2 (n = 6)	3 (n = 4)	2 (n = 4)	2 (n = 17)	0.48
Parasitaemia (parasites/µl), geometric mean (range)	36,450 (0–306,400)	40,311 (0–286,090)	34,479 (0–284,000)	35,564 (0–287,520)	36,640 (0–306,400)	0.87
Packed cell volume (%), mean ± SD	19.7 ± 7.4	21.0 ± 7.7	22.6 ± 6.9	20.3 ± 6.7	20.9 ± 7.2	0.15
Blood glucose (g/l), mean ± SD	1.03 ± 0.22	1.12 ± 0.29	1.06 ± 0.25	1.01 ± 0.20	1.05 ± 0.24	0.11
White blood cells (number/mm^3^), median (quartiles)	8000 (4200–23,200)	9400 (4900–26,800)	9600 (4800–19,000)	8650 (4500–22,800)	8700 (4200–26,800)	0.53

**Table 2:** Frequency of signs and symptoms of severity of malaria on admission according to the treatment regimens

Criteria of severity on admission	Regimens					p value
	ARTES (n = 59) n (%)	QLD (n = 57) n (%)	QNLD3 (n = 62) n (%)	QNLD2 (n = 60) n (%)	Total (n = 238) n (%)	
Hyperthermia (θ ≥ 40 °C)	42 (71.2)	39 (68.4)	52 (83.9)	49 (81.7)	182 (76.5)	0.12
Severe anaemia	28 (47.5)	24 (42.1)	20 (32.3)	16 (26.7)	88 (37.0)	0.08
Convulsions	10 (16.9)	19 (33.3)	20 (33.3)	17 (28.3)	66 (28.0)	0.16
Persistent vomiting	13 (22.0)	19 (33.3)	10 (16.1)	16 (26.7)	58 (24.4)	0.16
Prostration	14 (23.7)	12 (21.1)	8 (12.9)	10 (16.7)	44 (18.5)	0.43
Coma	3 (5.1)	6 (10.5)	4 (6.5)	4 (6.7)	17 (7.1)	0.95
Hyperparasitaemia	5 (8.5)	3 (5.3)	2 (3.2)	5 (8.3)	15 (6.3)	0.57
Respiratory distress	5 (8.5)	2 (3.5)	3 (4.8)	3 (5.0)	13 (5.5)	0.68
Abnormal behaviour	2 (3.4)	4 (7.0)	3 (4.8)	2 (3.3)	11 (4.6)	0.76
Jaundice	2 (3.4)	3 (5.3)	2 (3.2)	1 (1.7)	8 (3.4)	0.76

Hyperthermia (76.5 %), severe anaemia (37.0 %) and convulsions (28.0 %) were the predominant manifestations of severe malaria in this setting (Table 2).

### Clinical outcome

**Fig. 2:**
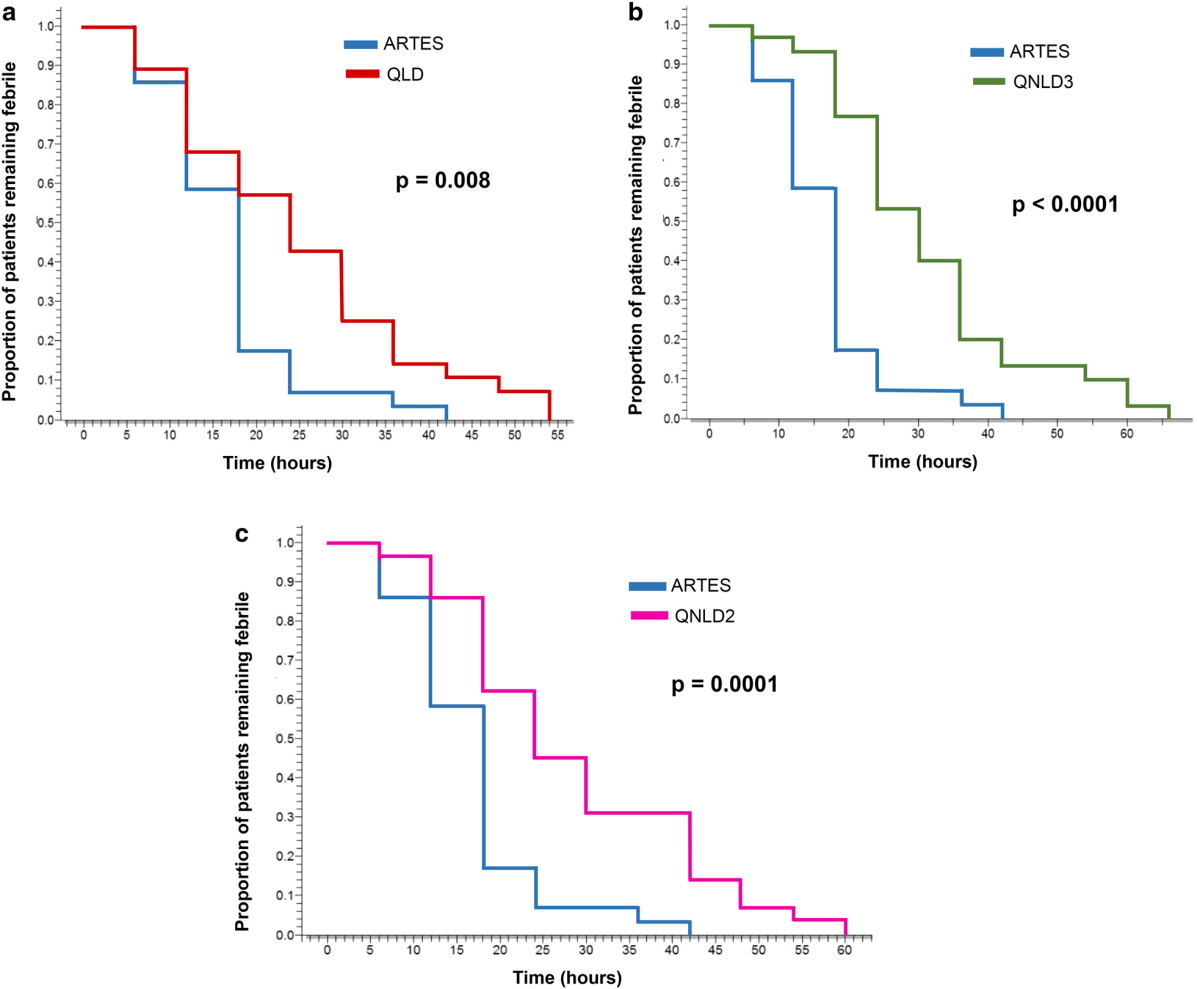
Kaplan-Meier plot of time to temperature <37.5 °C comparing. **a** Artesunate and the quinine loading dose regimen, **b** artesunate and the quinine non-loading dose three-daily administration regimen and **c** artesunate and the quinine non-loading dose two-daily administration regimen

On analysis of endpoints, the two-by-two comparison of artesunate with each quinine arm showed that fever clearance time, and time to exit from the protocol were significantly shorter in ARTES group than in the three quinine groups. Time to sit unsupported and time to eat were shorter for ARTES but the difference was not significant (Table 3). The survival curves for fever clearance are illustrated in Fig. 2. By comparing artesunate with each quinine curves, the Kaplan–Meier plots showed that the artesunate curve remained below the different quinine curves all through and attained a zero proportion of febrile patients faster than the three quinine curves with significant p-values obtained by Log-rank test. The overall time from onset of treatment to the last patient with fever was 42 h for ARTES, 54 h for QLD, 66 h for QNLD3 and 60 h for QNLD2.

**Table 3:** Clinical and parasitological outcome variables

Outcome variables	Regimens						
	ARTES (n = 29)	QLD (n = 28)		QNLD3 (n = 30)		QNLD2 (n = 29)	
	Mean ± SD	Mean ± SD	p value	Mean ± SD	p value	Mean ± SD	p value
Fever clearance time (hours)	16.8 ± 8.1	24.9 ± 13.9	*0.03*	31.2 ± 14.3	*<0.001*	28.6 ± 14.0	*<0.001*
Coma recovery time (hours)	40.5 ± 36.1 (n = 2)	45.0 ± 21.2 (n = 2)	1.0	30.0 ± 15.6 (n = 3)	1.0	36.0 ± 0.0 (n = 2)	1.0
Time to sit unsupported (hours)	7.7 ± 5.4 (n = 13)	13.9 ± 1.8 (n = 7)	0.7	15.9 ± 2.3 (n = 6)	0.4	17.3 ± 1.9 (n = 9)	0.7
Time to eat (hours)	4.1 ± 3.8 (n = 14)	6.2 ± 2.7 (n = 12)	0.1	8.7 ± 2.5 (n = 8)	0.07	11.3 ± 2.3 (n = 9)	0.06
Time to exit from the protocol (hours)	28.6 ± 10.3	34.3 ± 12.8	*0.01*	37.4 ± 17.3	*0.002*	36.7 ± 12.4	*0.01*
PCT (hours)	19.2 ± 11.3	29.3 ± 18.0	*0.03*	36.4 ± 21.2	*<0.001*	29.5 ± 19.2	*<0.001*
PRR_24_ (%)	92.0 ± 10.4 (n = 9)	74.8 ± 24.0 (n = 18)	*0.03*	66.5 ± 22.0 (n = 22)	*<0.001*	71.7 ± 23.7 (n = 22)	*0.03*

Significant p values are in italics

*PCT* Parasite clearance time, *PRR*_*24*_ Parasite reduction rate at H24

### Parasitological outcome

**Fig. 3:**
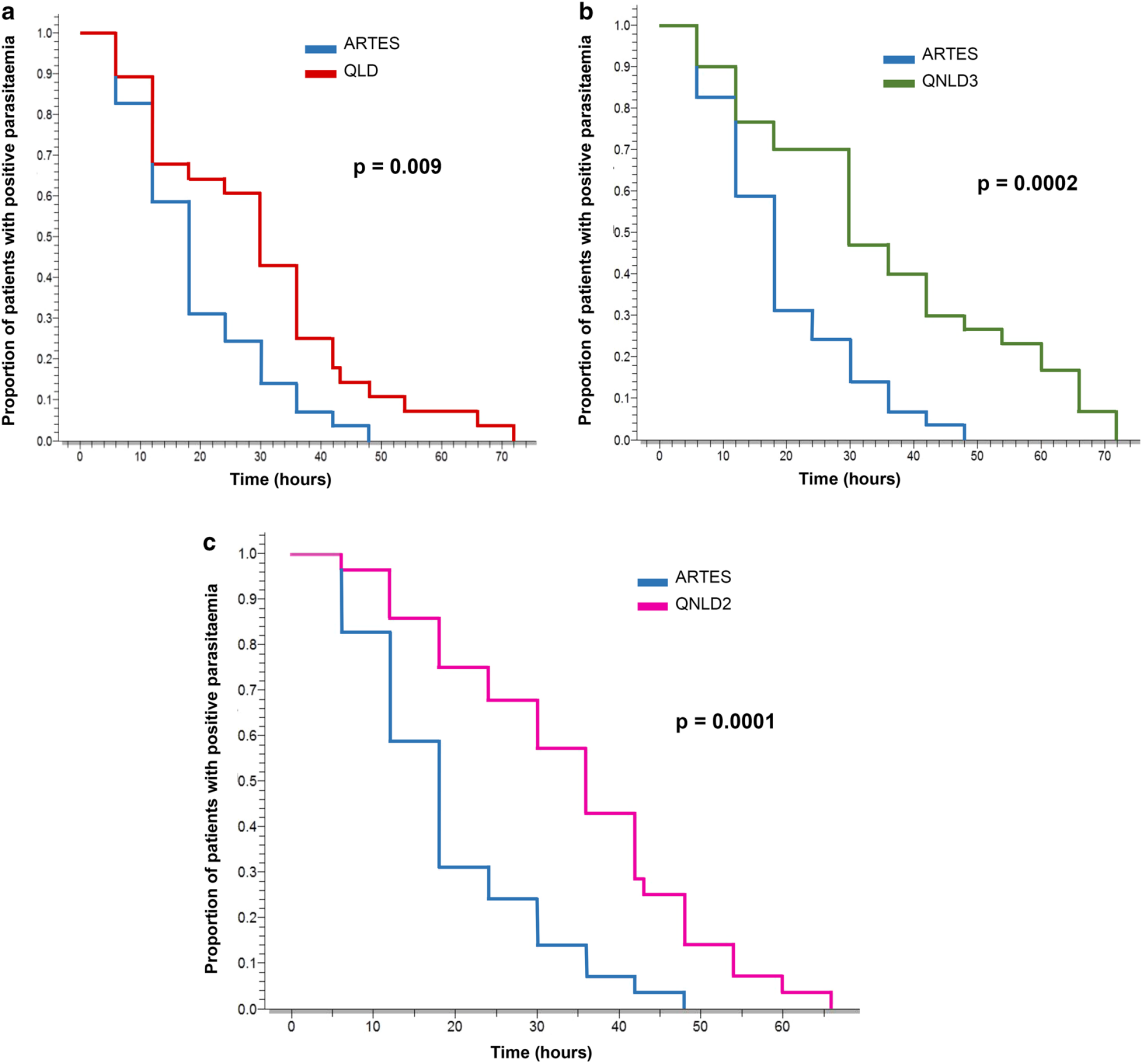
Kaplan-Meier plot of time to negative parasitaemia comparing. **a** Artesunate and the quinine loading dose regimen, **b** artesunate and the quinine non-loading dose three-daily administration regimen and **c** artesunate and the quinine non-loading dose two-daily administration regimen

The two-by-two comparison of artesunate with each quinine arm showed that parasite clearance time (PCT) was significantly shorter for artesunate than for the three quinine regimens. Parasite reduction rate 24 h after onset of treatment was significantly higher for patients in ARTES arm (92.0 ± 10.4 %) than for patients in QLD arm (74.8 ± 24.0 %, p = 0.03), QNLD3 arm (66.5 ± 22.0 %, p < 0.001), and QNLD2 arm (71.7 ± 23.7 %, p = 0.03) (Table 3). Kaplan–Meier survival curves of parasitaemia clearance comparing artesunate with each quinine regimen as illustrated in Fig. 3, showed that the artesunate curve remained below the different quinine curves all through and attained a zero proportion of parasitaemia faster than the three quinine curves with significant p values obtained by Log-rank test. The overall time from onset of treatment to the last patient with parasitaemia was 48 h for ARTES, 72 h for QLD, 72 h for QNLD3, and 66 h for QNLD2.

### Global outcome

There were two cases of adverse reactions warranting discontinuation of treatment. This was in the QLD and QNLD2 groups, in which one patient each developed mild urticaria and haemoglobinuria, respectively. In both cases, quinine was discontinued and artemether was instituted at a dose of 1.6 mg/kg/12 h on day 1, then at 1.6 mg/kg/24 h as from day 2. After complete cure, the patient who developed haemoglobinuria was referred to Yaounde for further investigations.

There was no neurological deficits on discharge of patients who were admitted with a coma or convulsions.

One patient died in ARTES group and in QNLD2 group representing a mortality rate of 1.7 % in each groups. The patient in the ARTES group was a 15 months old girl who was admitted with severe anaemia (packed cell volume = 8 %) and an episode of generalized tonico-clonic seizures lasting less than 30 min, following an eight-day history of fever. The patient had hyperparasitaemia at 285,300 TPf/µl and the CSF analysis was normal. The patient developed severe respiratory distress and died 4 hours after onset of treatment.

The patient in QNLD2 group was a 20 months old boy who was also admitted with severe anaemia (packed cell volume = 11 %) and two episodes of generalized tonico-clonic seizures lasting less than 30 min, following a 5-day history of fever. The patient also had hyperparasitaemia at 272,500 TPf/µl and the CSF analysis was normal. The patient went into coma and died 18 h after starting treatment.

## Discussion

This small sample size study reveals that artesunate is more effective than quinine in the treatment of severe malaria in Cameroonian children. Among artesunate-treated patients, fever clearance time, time to exit from the protocol and PCT were shorter; the parasite reduction rate 24 h after onset of treatment was higher than for quinine-treated patients. The four groups did not differ in time to sit unsupported, time to eat and CRT.

Fever clearance time (FCT) was shorter for patients in ARTES arm, followed by QLD arm, then QNLD2 arm, and finally QNLD3 arm. Considering ARTES as the reference arm, the two-by-two group comparison showed significant differences in FCT between artesunate and the three quinine groups. This is a consistent finding with studies comparing artesunate with the loading dose regimen of quinine [6–9]. This faster fever clearance with artesunate could be the result of its superior efficacy as compared to quinine. In this study, FCT in ARTES arm was 16.8 ± 8.1 h which is similar to 16.2 ± 8.9 h obtained by Hatim et al. in Sudanese children [7] and shorter than 43.6 ± 20.1 h reported by Mohanty et al. in Indian children [6]. In QLD group, FCT in this study was shorter than the 27.9 ± 21.4 h obtained by Forlack et al. [10] who used a loading dose of quinine followed later by a 12-hourly maintenance dose. This difference demonstrates the effectiveness of the current WHO recommendation. Furthermore, FCT was shorter for the group of patients who received a loading dose of quinine than for those who did not. This complies with pharmacokinetic studies, which suggests that a loading dose of quinine reduces the time to reach therapeutic plasma concentrations. Globally, FCT was shorter than PCT (25.4 ± 13.8 vs 31.8 ± 17.1 h). Rapid fever clearance is an important clinical benefit, but does not reflect cure and might create a false impression of cure, hence, the need for an oral relay to achieve complete cure. In this study, parenteral treatment was relayed with either artesunate-amodiaquine or artemether-lumefantrine for 3 days. Healthcare providers need to ensure that patients or caregivers understand the need for further curative treatment.

The CRT in ARTES arm was longer than in the QNLD3 and QNLD2 arms and shorter than in the QLD arm, but these differences were not statistically significant. The CRTs obtained for the four treatment groups were all higher than that observed by other authors [7, 8, 11–13]: Hatim et al. in Sudan for artesunate obtained a CRT of 8.1 h [7], 24 h for Ha Vinh et al. [11] and 32 h for Haroon et al. in adults [8]. Using the loading dose regimen of quinine, a CRT of 9.1 h was obtained in Sudan [7] and 12 h was observed in India [8]. When using the three-daily administration of quinine without a loading dose, Fargier et al. in Cameroon obtained a CRT of 13.0 h [12]. For the two-daily administration of quinine without a loading dose, a CRT of 28.6 ± 14.4 h was observed in Togo [13]. The lack of statistical significance in CRT between the treatment groups and the differences observed with other authors might be explained by the small number of patients with coma in this series. Haroon et al. [8] found intriguing the fact that the CRT was shorter in quinine-treated patients than in artesunate-treated patients despite shorter parasite clearance with artesunate and postulated that the neurotoxicity of artemisinin compounds might delay coma resolution. Contrarily, greater evidence of the efficacy of artesunate to reduce coma time faster than quinine has been shown by Dondorp et al. [14] in the African Quinine Artesunate Malaria Trial (AQUAMAT), where they observed less frequent development of coma (3.5 % with artesunate vs 5.1 % with quinine, p = 0.0231) and less deterioration of the coma score (6.1 % with artesunate vs 7.7 % with quinine, p = 0.0199) in artesunate recipients. Similar results were also obtained in the Southeast Asian Quinine Artesunate Malaria Trial (SEAQUAMAT). This benefit with artesunate might be due to the fact that artesunate inhibits the ability of parasitized red cells to stick onto endothelial cells (cyto-adherence) much more effectively than most other anti-malarials. Cyto-adherence is a recognized virulence determinant, therefore inhibiting it more efficiently may confer an advantage over other classes of anti-malarial drugs [15].

Time to sit unsupported and time to eat were shorter for artesunate-treated patients than for quinine-treated patients, but the differences were not significant. This is probably because of the small number of patients followed up for these variables. Dondorp et al. [14] also obtained a shorter time to sit unsupported and time to eat with artesunate than with quinine. This is possibly related to the rapid PCT observed in patients with artesunate.

Time to exit from the protocol was significantly shorter for artesunate-treated patients than for quinine-treated patients. This was likely due to the faster parasite clearance with artesunate, which leads to a faster clinical recovery.

PCT was significantly shorter for ARTES group as compared to QLD, QNLD3 and QNLD2 groups. Hatim et al. in Sudan [7] obtained a PCT for artesunate of 19.7 ± 7.1 h which is similar to that obtained in this study, whereas longer PCT has been reported in Asia: 52.2 ± 12.7 h in India [6] and 24 h in Vietnam [11]. These differences might be due to inter-individual variability. However, these authors also reported a shorter PCT with artesunate than with quinine. This shorter PCT is due to the fact that artemisinins (artesunate) act more rapidly in killing parasites and in inhibiting their major metabolic processes, such as glycolysis, nucleic acid and protein synthesis. It also attacks the broadest age range of parasites, from the tiniest rings that have recently invaded erythrocytes to more mature stages of parasites, such as developing trophozoites [15].

Parasite reduction rate 24 h after onset of treatment was significantly higher for patients in the ARTES arm. This information is important because, according to WHO, death from severe malaria often occurs within hours of admission to the hospital, so it is essential that therapeutic concentrations of a highly effective anti-malarial be achieved and maximum parasite clearance be obtained within the first 24 h [3].

### Limitations

Some weaknesses should be noted in this study. Firstly, the differences between the groups may have been underestimated because of the small sample size. Secondly, patients had to be naïve to the drugs used in the study but patients who took anti-malarials before admission to the hospital were included in the study because of time limitation and excluding them would have considerably reduced the sample size. However, most of the patients (40/54) who were treated with the study drugs to completion received anti-malarial drugs (quinine tablets or syrup or an artemisinin combination therapy (ACT)) at inadequate doses before admission. Thirdly, only short-term monitoring in the hospital during the hospital stay was done.

## Conclusion

This trial, in a population of Cameroonian children, shows that artesunate is more effective than quinine in the treatment of severe malaria. These results, together with those of the largest trials on artesunate (AQUAMAT and SEAQUAMAT), should reassure practitioners about the beneficial effects of artesunate over quinine and serve as strong motivation to prescribe artesunate, when available, as first-line drug to patients with severe malaria.
